# Circadian clock components RORα and Bmal1 mediate the anti-proliferative effect of MLN4924 in osteosarcoma cells

**DOI:** 10.18632/oncotarget.11807

**Published:** 2016-09-01

**Authors:** Shuju Zhang, Jiaming Zhang, Zhiyuan Deng, Huadie Liu, Wei Mao, Fang Jiang, Zanxian Xia, Jia-Da Li

**Affiliations:** ^1^ The State Key Laboratory of Medical Genetics and School of Life Sciences, Central South University, Changsha, Hunan 410078, China; ^2^ Xiangya Stomatological Hospital, Central South University, Changsha, Hunan 410078, China

**Keywords:** MLN4924, osteosarcoma, RORα, Bmal1, neddylation

## Abstract

The anticancer small molecule MLN4924, a Nedd8-activating enzyme (NAE) inhibitor, triggers cell-cycle arrest, apoptosis, and senescence in cancer cells. In this study, we demonstrate that MLN4924 suppresses osteosarcoma cell proliferation by inducing G2/M cell cycle arrest and apoptosis. Our results indicate that MLN4924 stabilizes the retinoid orphan nuclear receptor alpha (RORα) by decreasing its ubiquitination. RNA interference of RORα attenuates the anti-proliferative effect of MLN4924 in U2OS osteosarcoma cells. MLN4924 up-regulates the expression of p21 and Bmal1, two transcriptional targets of RORα. However, p21 plays a minimal role in the anti-proliferative effect of MLN4924 in U2OS osteosarcoma cells. In contrast, Bmal1 suppression by siRNA attenuates the anti-proliferative effect of MLN4924 in U2OS osteosarcoma cells, indicating that the MLN4924-mediated cell growth inhibition is mediated by Bmal1. These results show MLN4924 to be a promising therapeutic agent for the treatment of osteosarcoma and suggest that MLN4924-induced tumor growth inhibition is mediated by the circadian clock components RORα and Bmal1.

## INTRODUCTION

Osteosarcoma is the most common primary malignant bone tumor in children and adolescents. In the past four decades, osteosarcoma has been treated with neoadjuvant chemotherapy followed by surgical removal of the primary tumor, often followed by additional adjuvant chemotherapy after the surgery. Cis-platinum, doxorubicin, methotrexate and cyclophosphamide have been used most often to treat osteosarcoma. Nevertheless, the 5-year overall survival rate of osteosarcoma is one of the lowest in the pediatric cancers, i.e. ∼65% for localized tumor [1, 2]. It is therefore necessary and urgent to identify new therapeutic strategies for osteosarcoma patients.

Nedd8 is a ubiquitin-like molecule of ∼8 kDa that is covalently linked to a number of proteins by a process known as neddylation. The most well characterized substrates of Nedd8 are the cullin proteins, the scaffold components of Cullin-Ring E3-ubiquitin Ligases (CRLs) that are responsible for ubiquitination of ∼20% of cellular proteins degraded through the ubiquitin-proteasome system [3]. MLN4924 (pevonedistat), a selective Nedd8 activating enzyme (NAE) inhibitor, is an anti-cancer drug currently in clinical trials [4, 5]. Treatment with MLN4924 can suppress the progression of a variety of tumors [6–14]. Various CRLs substrates have been proposed to mediate the anti-cancer effects of MLN4924 [5, 15–17].

In this study, we show that MLN4924 suppresses the proliferation of osteosarcoma cells, which may be mediated, at least partly, through the retinoid orphan nuclear receptor alpha (RORα).

## RESULTS

### MLN4924 suppresses growth and tumorigenicity of osteosarcoma cells

**Figure 1:**
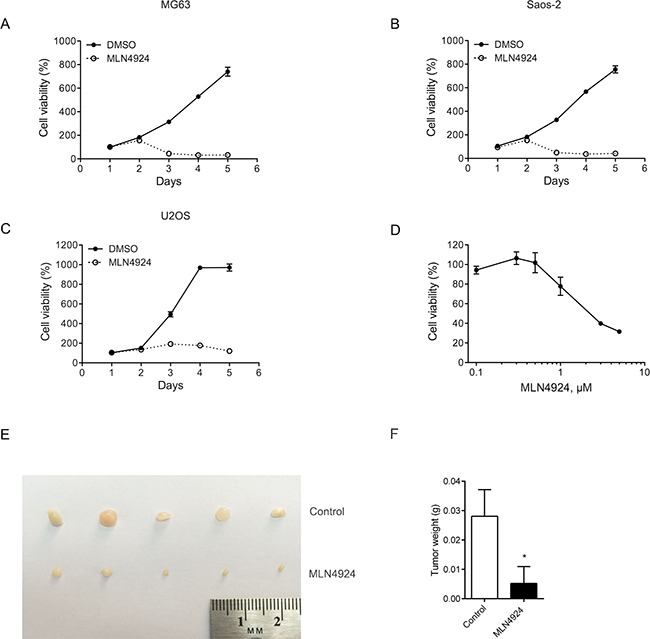
MLN4924 inhibits cell growth and tumorigenicity of osteosarcoma cells **A-C.** Three osteosarcoma cell lines: MG63, Saos-2 and U2OS were treated with MLN4924 (3 μM) or DMSO. Cell viability was assessed by an MTT assay. Each value is the mean ± SEM of three replicates from a single assay. **D.** U2OS cells were treated with various concentrations of MLN4924 for 48 h. Cell viability was assessed by MTT assay. **E.** MLN4924 significantly inhibited the growth of MG63 cells xenografts *in vivo*. 1 × 10^6^ MG63 cells were injected subcutaneously into the flanks of nude mice. Ten days later, mice were treated with vehicle (control) or MLN4924 (30 mg/kg i.p.) twice a day for 3 days, and then 2 days without treatment for 15 days. **F.** Tumor weights after 15 days of treatment. *P < 0.05, unpaired *t* test.

We first evaluated the effect of MLN4924 on cell proliferation in three osteosarcoma cell lines: MG63, Saos-2 and U2OS. As shown in Figure 1A-1C, one day of MLN4924 (3 μM) treatment did not significantly change the cell growth; however, MLN4924 significantly inhibited the cell proliferation after two days treatment and thereafter. In U2OS cells, MLN4924 suppressed cell growth in a dose-dependent manner (Figure 1D).

We next determined the anti-tumor activity of MLN4924 in osteosarcoma cells *in vivo*. MLN4924 was administered to nude mice bearing MG63 xenografts; the tumors were weighted after 15 days of the treatment. As shown in Figure 1E and 1F, MLN4924 significantly decreased the growth of MG63 xenograft tumors.

**Figure 2:**
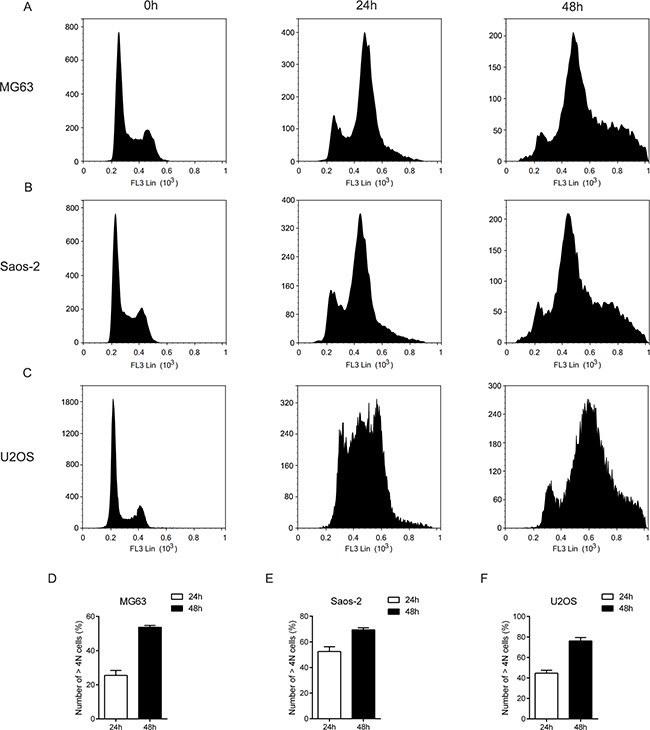
MLN4924 causes G2/M cell cycle arrest in osteosarcoma cells Three osteosarcoma cell lines: MG63 **A.**, Saos-2 **B.** and U2OS **C.** were treated with DMSO or MLN4924 (1 μM) for 24 and 48 h. Cells were harvested and fixed in ice-cold 70% ethanol overnight at −20°C, and then stained with PI (5 μg/100 μL) for 30 min at 4 °C in the dark. DNA profiles were analyzed by flow cytometry. > 4*N* cells were shown in **D-F.** Each value was the mean ± SEM of three replicates from a single assay.

To determine how MLN4924 affects the cell cycle, a flow cytometry analysis was performed in three osteosarcoma cell lines: MG63, Saos-2 and U2OS in the presence of MLN4924. As shown in Figure 2, after 24 h treatment with MLN4924 (1 μM), cells began to accumulate in G2/M phase. At 48 h after MLN4924 treatment, the cells with more than 4 chromosomes (≥ 4 *N* DNA content) were significantly increased; this was similar to the MLN4924 effect in HCT116 cells [5].

**Figure 3:**
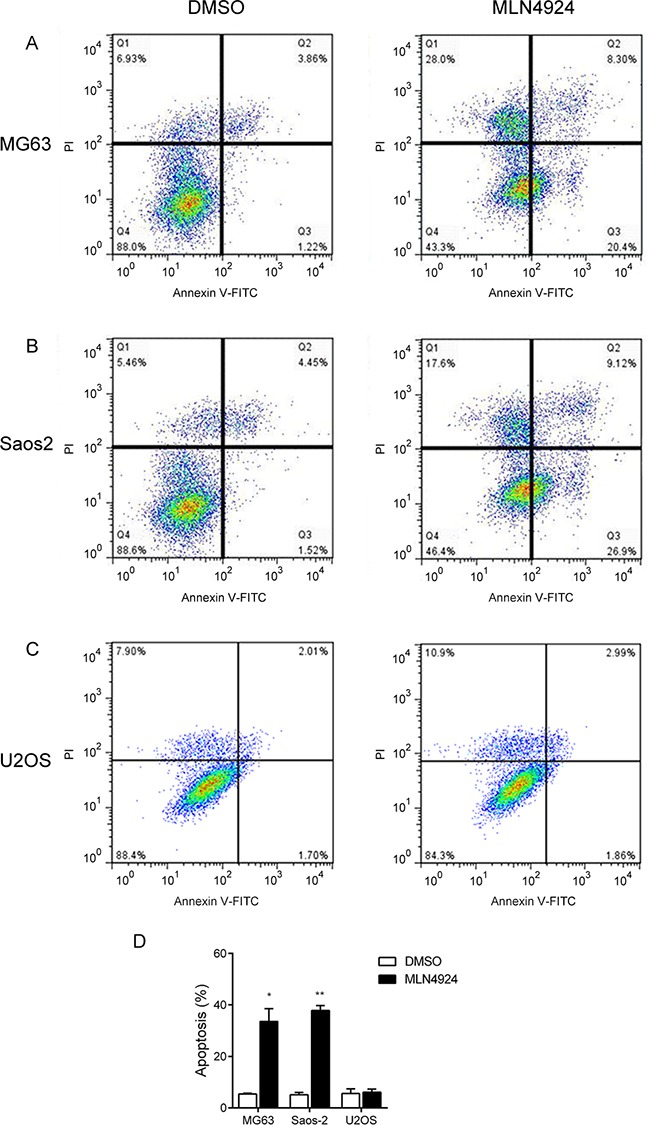
MLN4924 induces apoptosis in MG63 and Saos-2, but not U2OS cells **A-C.** Three osteosarcoma cell lines MG63 (A), Saos-2 (B) and U2OS (C) were treated with DMSO or MLN4924 (1 μM) for 48 h. Cells were harvested and stained with Annexin V-FITC and PI for 20 min in the dark. Apoptosis was analyzed by flow cytometry. **D.** The graph illustrates the percentage of total apoptosis cells. Each value was the mean ± SEM of three replicates from a single assay. Q1: live cells (annexin V^−^/PI^−^), Q2: early apoptotic cells (annexin V^+^/PI^−^), Q3: late apoptotic cells (annexin V^+^/PI^+^) and Q4:necrotic cells (annexin V^−^/PI^+^).*P < 0.05, **P < 0.01 unpaired *t* test.

We also investigated the apoptotic effect of MLN4924 in the osteosarcoma cell lines. After labelling with Annexin V-FITC/PI, a flow cytometry was performed to analyze the apoptotic cells. As shown in Figure 3, treatment with MLN4924 (1 μM) for 48 h induced significant apoptosis in MG63 and Saos-2 cells, but not in U2OS cells. (Apoptotic cells: MG63, DMSO: 5.37% ± 0.29, MLN4924: 33.60% ± 4.90, *P* = 0.003; Saos-2, DMSO: 5.08% ± 0.89, MLN4924: 37.89% ± 2.07, *P* = 0.004; U2OS, DMSO: 5.60% ±1.81, MLN4924: 6.10% ± 1.25, *P* = 0.84, Figure 3D)

### MLN4924 increases stability of RORα

The retinoid orphan nuclear receptor alpha (RORα) is an orphan nuclear receptor that regulates gene expression by binding to the ROR response elements (RORE). Recent studies indicate that RORα functions as a tumor suppressive molecule [18]. Interestingly, RORα is degraded by the DCAF1/DDB1/CUL4 E3 ubiquitin ligase complex [19, 20], which might be inhibited by MLN4924. We have therefore reasoned that RORα may mediate the effect of MLN4924.

**Figure 4:**
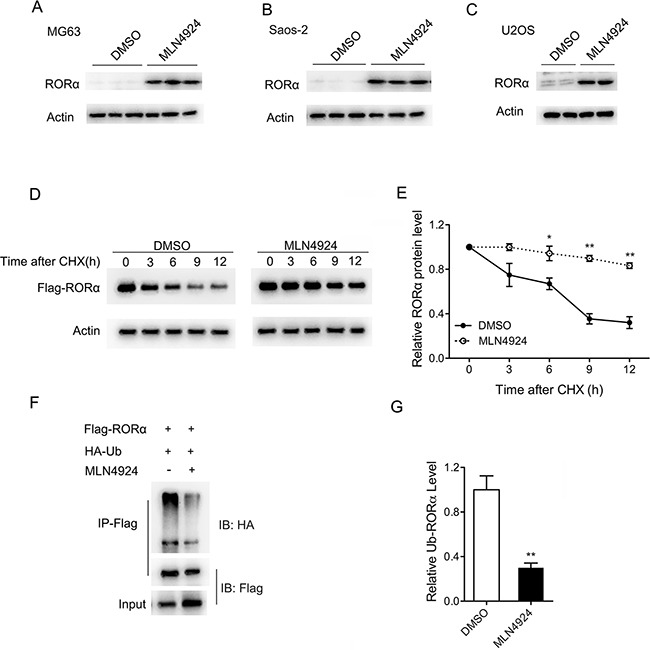
MLN4924 increases the stability of RORα **A-C.** The endogenous RORα protein levels detected with Western blot after treatment with MLN4924 (1 μM) or DMSO in MG63 (A), Saos-2 (B) and U2OS (C) cells for 24 h. **D.** MLN4924 increased the half-life of RORα. U2OS cells were transiently transfected with plasmids expressing the Flag-RORα. At 24 h after transfection, MLN4924 (1 μM) or DMSO were added into respective cell culture media. 24 h later, cells were treated with cycloheximide (CHX) for 0, 3, 6, 4, 9 and 12 h. Equal amounts of whole cell lysates were analyzed by Western blot with a Flag antibody (M2). Actin was used as an internal control. **E.** The graph illustrates the quantification of RORα by densitometry of triplicate experiments (mean ± SEM). *P < 0.05, **P < 0.01 by *post hoc* Bonferroni *t* test. **F.** MLN4924 decreased the ubiquitination of RORα. Flag-RORα and HA-Ub expression plasmids were transiently transfected into U2OS cells. At 24 h after transfection, MLN4924 (1 μM) or DMSO were added into respective cell culture media. 24 h later, cells were treated with MG132 for 8 h. Equal amounts of whole cell lysates were used for immunoprecipitation (IP). The protein immunoprecipitated by Flag antibody was analyzed by Western blot with HA or Flag antibody. **G.** The graph illustrates the quantification of ubiquitinated RORα by densitometry of triplicate experiments (mean ± SEM). **P < 0.01 by unpaired *t* test.

To investigate whether MLN4924 affects the degradation of RORα, we first examined the endogenous RORα protein levels in osteosarcoma cells treated 24 h with MLN4924. As shown in Figure 4A-4C, RORα was significantly up-regulated in osteosarcoma MG63, Saos-2, and U2OS cells after MLN4924 (1 μM) treatment.

To further investigate if MLN4924 affects the stability of RORα, U2OS cells transiently expressing Flag-labelled RORα (Flag-RORα) were treated 24 h with MLN4924 (1 μM) or DMSO. 48 hours after transfection, cells were incubated 0, 3, 6, 9, and 12 h with cycloheximide (CHX) to inhibit new protein synthesis, and RORα protein levels were analyzed. As shown in Figure 4D and 4E, MLN4924 significantly prolonged the half life of RORα. Without MLN4924, the half life of RORα was about 7 h, whereas only about 20% of RORα was degraded after 12 h in the presence of MLN4924.

Next, we studied the effect of MLN4924 on the ubiquitination of RORα. U2OS cells co-expressing Flag-RORα and HA-tagged uniquitin (HA-Ub) were treated with MLN4924 (1 μM) or DMSO at 24 h after transfection. Cells were incubated 8 h with proteasome inhibitor MG-132 (10 μM) 48 h after transfection, and immunoprecipitation was performed. The anti-Flag antibody was used to pulldown Flag-RORα proteins, and ubiqutination was detected with the anti-HA antibody. As shown in Figure 4F and 4G, the ubiquitination of RORα was decreased by about 70% in the presence of MLN4924.

### Suppression of ROR α attenuates MLN4924-induced cell cycle arrest

**Figure 5:**
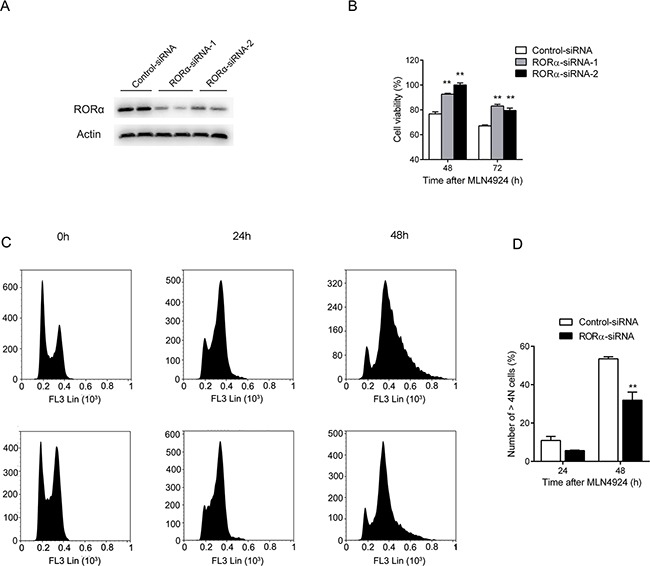
Down-regulation of RORα attenuates MLN4924-induced cell growth suppression **A.** U2OS osteosarcoma cells were transfected with two distinct RORα-specific siRNAs or a negative control siRNA. 72 h after transfection, whole cell lysates were analyzed with Western blot using an antibody against RORα. **B.** U2OS cells were treated with MLN4924 (3 μM) or DMSO for 48 h after transfection with RORα-specific siRNAs or negative control siRNA, and the cell proliferation was monitored with an MTT assay at 48 and 72 h after MLN4924 treatment. Each value is the mean ± SEM of three replicates from a single assay (**P < 0.01 by *post hoc* Bonferroni *t* test). **C.** U2OS cells transfected with RORα-specific siRNA or negative control siRNA after MLN4924 (1 μM) treatment for 0, 24 and 48 h. Cells were harvested and fixed in ice-cold 70% ethanol overnight at −20°C, then stained with PI (5 μg/100 μL) for 30 min at 4 °C in the dark. DNA profiles were analyzed by flow cytometry. **D.** The graph illustrates the percentage of > 4*N* cell of four replicates from a single assay (**P < 0.01 by *post hoc* Bonferroni *t* test).

To determine whether MLN4924 acts through RORα to suppress cell growth, U2OS osteosarcoma cells were transfected with two distinct RORα-specific siRNAs (Figure 5A). The cells were treated 48 and 72 h with MLN4924 (3 μM) or DMSO at 48 h after transfection, and the cell proliferation was monitored with an MTT assay. Compared with cells transfected with a negative control siRNA, MLN4924 was significantly less effective in suppressing the growth of RORα-depleted cells (Cell viability: 48 h, Control: 76.74% ± 1.72, RORα-siRNA-1: 92.62% ± 0.76, *P* < 0.0001, RORα-siRNA-2: 100.0% ± 1.69, *P* < 0.0001; 72 h, Control: 67.08% ± 0.80, RORα-siRNA-1: 83.12% ± 1.39, *P* < 0.0001, RORα-siRNA-2: 79.47% ± 1.97, *P* < 0.0002, Figure 5B).

To further examine whether RORα participated in MLN4924-induced cell cycle arrest, flow cytometry analysis was performed on U2OS cells transfected with control or RORα-specific siRNA-1 after MLN4924 (1 μM) treatment for 0, 24 and 48 h. As shown in Figure 5C-5D, RORα suppression significantly attenuated the G2/M cell cycle arrest after 48h treatment, as illustrated with percentage of cells at > *4N* stages (24 h, Control: 10.91% ± 1.50, RORα-siRNA: 5.61% ± 0.18, *P* = 0.33; 48 h, Control: 53.47% ± 0.80, RORα-siRNA: 31.95% ± 2.95, *P* = 0.02).

### p21 minimally affects the MLN4924-induced cell growth suppression

**Figure 6:**
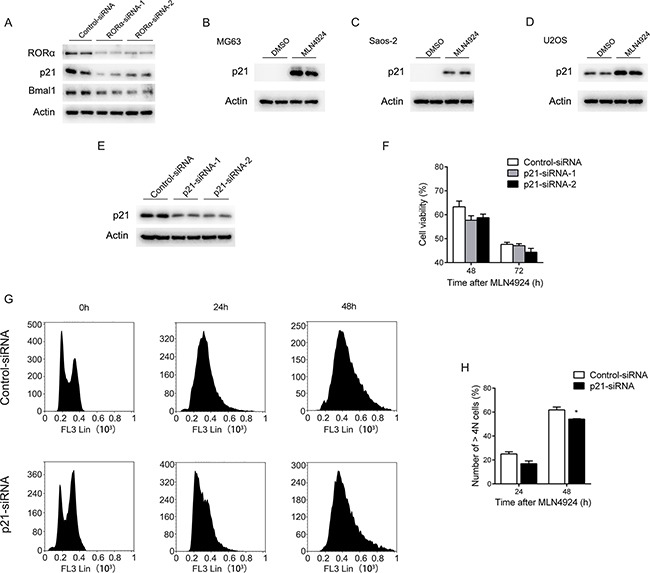
p21 minimally affects the MLN4924-induced cell growth inhibition **A.** U2OS osteosarcoma cells were transfected with two distinct RORα-specific siRNAs or a negative control siRNA. 72 h after transfection, whole cell lysates were analyzed with Western blot using antibodies for RORα, p21, Bmal1 and actin, respectively. **B-D.** The endogenous p21 protein levels detected with Western blot after treatment with MLN4924 (1 μM) or DMSO in MG63 (B), Saos-2 (C) and U2OS (D) cells for 24 h. **E.** U2OS osteosarcoma cells were transfected with two distinct p21-specific siRNAs or a negative control siRNA. 72 h after transfection, whole cell lysates were analyzed with Western blot using an antibody against p21. **F.** U2OS cells were treated with MLN4924 (3 μM) or DMSO from 48 h after transfection with p21-specific siRNA or negative control siRNA, and the cell proliferation was monitored with an MTT assay at 48 and 72 h after MLN4924 treatment. Each value is the mean ± SEM of three replicates from a single assay. **G.** U2OS cells transfected with p21-specific siRNA or negative control siRNA after MLN4924 (1 μM) treatment for 0, 24 and 48 h. Cells were harvested and fixed in ice-cold 70% ethanol overnight at −20°C, then stained with PI (5 μg/100 μL) for 30 min at 4 °C in the dark. DNA profiles were analyzed by flow cytometry. (D) The graph illustrates the percentage of > 4*N* cell of four replicates from a single assay (*P < 0.05 by *post hoc* Bonferroni *t* test).

Next, we investigated the downstream molecules of RORα involved in the MLN4924 anti-cancer effect. RORα is a putative transcription factor for cyclin-dependent kinase (CDK) inhibitor p21WAF1/CIP1. Indeed, RNAi of RORα resulted in downregulation of p21 (Figure 6A). In addition, p21 was up-regulated in osteosarcoma cells MG63, Saos-2, and U2OS after MLN4924 (1 μM) treatment (Figure 6B-6D).

To determine whether p21 is involved in the MLN4924-induced cell growth suppression, U2OS cells were transfected with two distinct p21-specific siRNAs (Figure 6E). Although both siRNAs significantly decreased the p21 levels, no significant effect was observed on the cell growth suppression elicited by MLN4924 (3 μM) (Cell viability: 48 h, Control: 63.32% ± 2.41, p21-siRNA-1: 57.68% ± 1.74, *P* = 0.071, p21-siRNA-2: 58.80% ± 1.34, *P* = 0.11; 72 h, Control: 47.60% ± 0.89, p21-siRNA-1: 47.10% ± 0.82, *P* = 0.68, p21-siRNA-2: 44.35% ± 1.59, *P* = 0.08, Figure 6F).

Then, we performed flow cytometry analysis of U2OS cells transfected with control or p21-specific siRNA-1 after MLN4924 (1 μM) treatment for 0, 24 and 48 h. As shown in Figure 6G and H, p21 suppression had a minimal effect on the MLN4924-induced G2/M accumulation and increase in the > *4N* cells (24 h, Control: 25.01% ± 1.29, p21-siRNA: 16.87% ± 1.65, *P* = 0.06; 48 h, Control: 61.84% ± 1.71, p21-siRNA: 54.16% ± 0.25, *P* = 0.05).

### MLN4924 increases Bmal1 expression at the transcriptional level

RORα induces the expression of Bmal1, the essential component of circadian clock. As expected, RNAi of RORα resulted in downregulation of Bmal1 in the absence or presence of MLN4924 (Figure 6A and Supplementary Figure S1). Recently, Bmal1 has been demonstrated to suppress cell growth through G2/M cell cycle arrest [21–24], suggesting that Bmal1 functions downstream of the MLN4924-RORα pathway.

**Figure 7:**
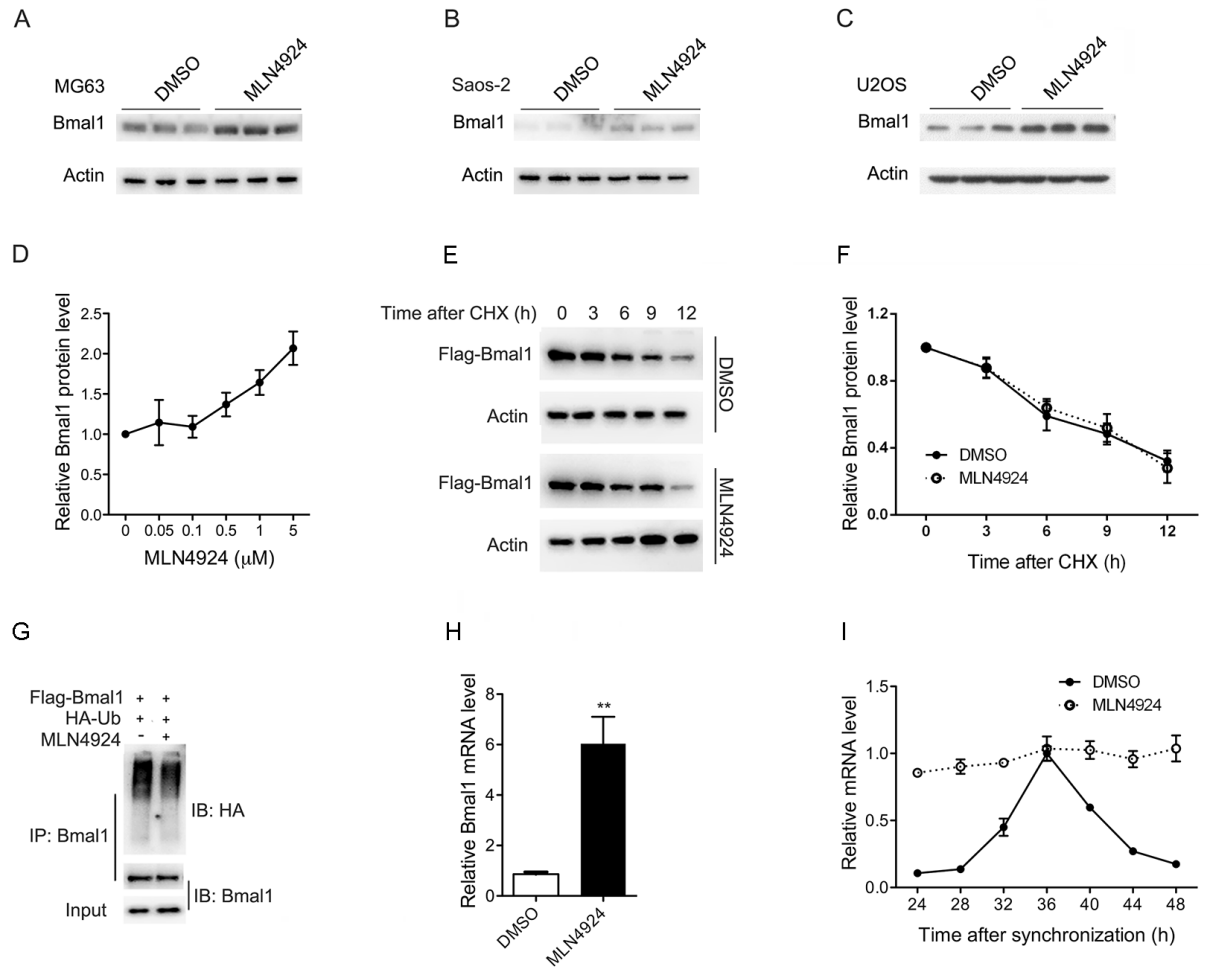
MLN4924 increases Bmal1 at the transcriptional level **A-C.** The endogenous Bmal1 protein levels detected with Western blot after treatment with MLN4924 (1 μM) or DMSO in MG63 (A), Saos-2 (B) and U2OS (C) cells for 24 h. **D.** U2OS cells treated with various concentration of MLN4924 for 24 h. Whole cell lysates were analyzed by Western blot using an antibody against Bmal1. The densitometry of Bmal1 treated without MLN4924 was set as 1. Data from various concentration of MLN4924 are presented as fold of this level. **E.** MLN4924 did not influence the half-life of Bmal1. U2OS cells were transiently transfected with plasmids expressing Bmal1. At 24 h after transfection, MLN4924 (1 μM) or DMSO were added into respective cell culture medium. 24 h later, cells were treated with cycloheximide (CHX) for 0, 3, 6, 9 and 12 h. Equal amounts of whole cell lysates were analyzed by Western blot with a Flag antibody. Actin was used as an internal control. **F.** The graph illustrates the quantification of Bmal1 by densitometry of triplicate experiments (mean ± SEM). **G.** MLN4924 did not influence the ubiquitination of Bmal1. Bmal1 and HA-Ub expression plasmids were transiently transfected into U2OS cells. At 24 h after transfection, MLN4924 or DMSO were added into respective cell culture medium. 24 h later, cells were treated with MG132 for 8 h. Equal amounts of whole cell lysates were used for immunoprecipitation (IP). The protein immunoprecipitated by Bmal1 antibody was analyzed by Western blot with HA and Bmal1 antibody. **H.** MLN4924 increased the Bmal1 transcription. U2OS cells were treated with MLN4924 (1 μM) or DMSO for 24 h, and the total RNA was extracted. The Bmal1 mRNA level was examined with qPCR using specific primers. The expression of Bmal1 in cells treated with DMSO was set as 1. Data from MLN4924-treated cell were presented as fold of this level. Each value is the mean ± SEM of three replicates from a single assay (**P < 0.01 by unpaired *t* test). **I.** U2OS cells were synchronized with dexamethasone in the presence of MLN4924 (1 μM) or DMSO, and then harvested every 4 h beginning 24 h after synchronization. The Bmal1 mRNA level was examined with qPCR using specific primers. The expression of Bmal1 in cells treated with DMSO at 36 h was set as 1. Data from other treatments were presented as fold of this level. Each value is the mean ± SEM of three replicates form a single assay. Solid line, DMSO; dashed line, MLN4924.

To investigate the role of Bmal1 in the anti-cancer effect of MLN4924, we first examined the endogenous Bmal1 protein levels after MLN4924 (1 μM, 24 h) treatment in osteosarcoma cell lines. As shown in Figure 7A-7C, Bmal1 was significantly up-regulated in osteosarcoma cells MG63, Saos-2, and U2OS after MLN4924 treatment. In U2OS osteosarcoma cells, Bmal1 protein levels were induced by MLN4924 in a concentration-dependent manner. (Figure 7D). Nevertheless, MLN4924 did not influence the half-life and ubiquitination of Bmal1 protein (Figure 7E-7G).

We therefore examined the effect of MLN4924 on Bmal1 transcription. As shown in Figure 7H, MLN4924 (1 μM) significantly up-regulated the mRNA level of Bmal1. We further examined the effect of MLN4924 on the rhythmic expression of Bmal1. U2OS cells were synchonization with dexamethasone in the presence or absence of MLN4924 (1 μM), and then harvested every 4 h beginning 24 h after synchonization. In the absence of MLN4924, Bmal1 expression showed a circadian rhythm, peaking at 36 h after snchronization. MLN4924 disrupted the circadian rhythm; Bmal1 expression was increased in all time points in the presence of MLN4924 (Figure 7I). It should be noted that both RORα mRNA and protein levels were significantly elevated under synchronized conditions (Supplementary Figure S2).

### Bmal1 participates in the MLN4924-induced cell growth inhibition

**Figure 8:**
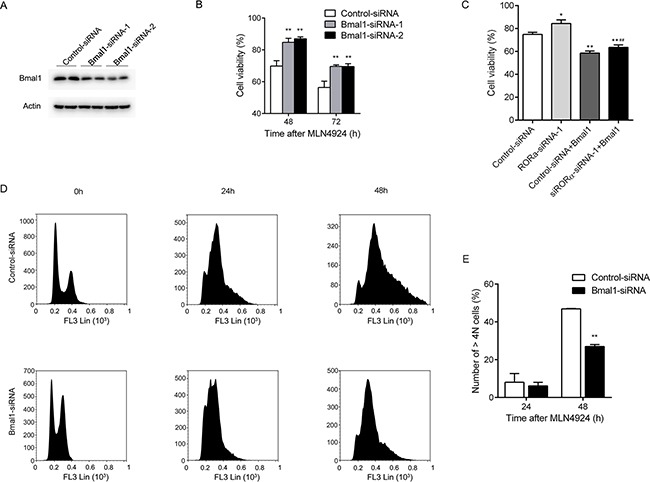
Bmal1 participates in the MLN4924-induced cell growth inhibition **A.** U2OS osteosarcoma cells were transfected with two distinct Bmal1-specific siRNAs or a negative control siRNA. 72 h after transfection, whole cell lysates were analyzed by Western blot using an antibody against Bmal1. **B.** U2OS cells were treated with MLN4924 (3 μM) or DMSO at 48 h after transfection with Bmal1-specific siRNAs or negative control siRNA, and the cell proliferation was monitored with an MTT assay at 48 and 72 h after MLN4924 treatment. Each value is the mean ± SEM of three replicates form a single assay. **C.** U2OS cells were treated with MLN4924 (3 μM) or DMSO at 48 h after transfection with RORα-specific siRNA or/and Bmal1-expressing plasmid, and the cell proliferation was monitored with MTT assay at 48 h after MLN4924 treatment. Each value is the mean ± SEM of three replicates from a single assay. (* compared with control group; # compared with RORα-siRNA-1 group. *P < 0.05, **P< 0.01, ##*P*< 0.01 by *post hoc* Bonferroni *t* test). **D.** U2OS cells transfected with Bmal1-specific siRNA or negative control siRNA were treated with MLN4924 (1 μM) for 0, 24 and 48 h. Cells were harvested and fixed in ice-cold 70% ethanol overnight at −20°C, then stained with PI (5 μg/100 μL) for 30 min at 4 °C in the dark. DNA profiles were analyzed by flow cytometry. **E.** The graph illustrates the percentage of > 4*N* cell of four replicates from a single assay (**P < 0.01 by *post hoc* Bonferroni *t* test).

To determine whether MLN4924 acts through Bmal1 to suppress cell growth, U2OS osteosarcoma cells were transfected with two distinct Bmal1-specific siRNAs (Figure 8A). The cells were treated 48 and 72 h with MLN4924 (3 μM) or DMSO at 48 h after transfection, and the cell proliferation was monitored with an MTT assay. Compared with cells transfected with control siRNA, MLN4924 was significantly less effective in suppressing the growth of Bmal1-depleted cells (cell viability: 48 h, Control: 69.83% ± 3.41, Bmal1-siRNA-1: 84.73% ± 2.56, *P =* 0.0013, Bmal1-siRNA-2: 86.99% ± 1.21, *P* < 0.0001; 72 h, Control: 56.37% ± 3.98, Bmal1-siRNA-1: 69.57%± 0.99, *P =* 0.0028, Bmal1-siRNA-2: 69.50% ± 1.82, *P =* 0.005, Figure 8B).

We also overexpressed Bmal1 in U2OS cells, and the anti-proliferative efficacy of MLN4924 was analyzed with MTT assay. As shown in Figure 8C, overexpression of Bmal1 significantly enhanced the effect of MLN4924 in both control and RORα-depleted U2OS cells. (Cell viability: Control: 74.80% ± 1.88, RORα-siRNA: 84.40% ± 3.17, Control+Bmal1: 58.5% ± 1.91, RORα-siRNA+Bmal1: 63.5% ± 2.13).

To further examine whether Bmal1 participates in MLN4924-induced cell cycle distribution, flow cytometry analysis was performed in U2OS cells transfected with control or Bmal1-specific siRNA-1 after MLN4924 (1 μM) treatment for 0, 24 and 48 h. As shown in Figure 8D-8E, Bmal1 depletion significantly attenuated the G2/M cell cycle arrest after 48h treatment, as illustrated with percentage of cells at > *4N* stages (24 h, Control: 8.09% ± 3.24, Bmal1-siRNA: 6.12% ± 1.33, *P* = 0.63; 48 h, Control: 46.89% ± 0.13, Bmal1-siRNA: 26.96% ± 0.75, *P* = 0.0014).

## DISCUSSION

In this study, we have shown that a Nedd8-activating enzyme inhibitor, MLN4924, suppresses proliferation of osteosarcoma cells through G2/M cell cycle arrest and apoptosis. In addition, we have demonstrated that the circadian clock components RORα and Bmal1 play important roles in the MLN4924 effect in osteosarcoma cells. MLN4924 stabilizes a cullin substrate RORα, which in turn transactivates Bmal1. Downregulation of RORα or Bmal1 in U2OS osteosarcoma cells attenuates the MLN4924-induced cell growth suppression as well as G2/M cell cycle arrest.

Physiology and behavior are subjected to daily oscillations driven by an endogenous circadian clock. Disruption of the circadian rhythm may lead to a series of disorders, including cancer. A study that followed 78,562 nurses showed that the nurses who always worked night shift for at least thirty years had a significantly increased risk of breast cancer as compared with those who only worked during daylight [25–27]. Accumulative evidence has shown that Bmal1 and RORα are tumor suppressors. Jiang et al have demonstrated that Bmal1 level is downregulated in pancreatic cancer [21]. Bmal1 is transcriptionally silenced by promoter CpG island hypermethylation in hematologic malignancies, such as diffuse large B-cell lymphoma and acute lymphocytic and myeloid leukemia [28]. Overexpression of Bmal1 inhibits, whereas RNA interference of Bmal1 promotes tumor cell proliferation as well as cell invasion. Mechanistically, overexpression of Bmal1 induces G2/M cell cycle arrest [22, 24]. Baek and colleagues have demonstrated that RORα is downregulated in breast cancer [19]. They also found a reduction of RORα phosphorylation in human colorectal tissues [29]. Moreover, Xu and colleagues have demonstrated that RORα suppresses breast tumor proliferation and invasion [30].

In this study, we have demonstrated that MLN4924 decreases the ubiquitination of RORα and subsequently its degradation in osteosarcoma cells. Although p21 is transcriptionally regulated by RORα, and p21 was indeed upregulated after MLN4924 treatment in U2OS osteosarcoma cells, p21 does not seem to play a significant role in the effect of MLN4924 in U2OS cells. Nevertheless, p21 may play a role in the effect of MLN4924 in MG63 and SaoS-2 osteosarcoma cells, considering that MLN4924 induced apoptosis in MG63 and SaoS-2, but not in U2OS cells.

We have further demonstrated that Bmal1, another transcriptional target of RORα, plays a critical role in MLN4924-induced cell growth suppression. Bmal1 is able to transactivate Wee1, which may in turn inhibit cyclin B/CDK1 to halt cells at G2/M cell cycle [31]. Indeed, MLN4924 significantly upregulated Wee1 and RNAi of RORα led to significant reduction in Wee1 (Supplementary Figure S3).

In summary, this study provides a rationale for clinical trials of MLN4924 in osteosarcoma. Our data indicate an important role of circadian clock in the anti-cancer effect of MLN4924 in osteosarcoma cells. As RORα and Bmal1 oscillate in a circadian manner, it will be intriguing to determine if the effect of MLN4924 is dependent on the circadian rhythm.

## MATERIALS AND METHODS

### Cell culture

Human osteosarcoma cell lines (U2OS, MG63 and Saos-2) were grown at 37 °C under 5% CO_2_ in Dulbecco's modified Eagle medium (DMEM) supplemented with 10% fetal bovine serum (FBS) and 100 U/mL of penicillin/streptomycin.

### Antibodies and reagents

Antibodies against Bmal1 (sc-8550) and RORα (sc-28612) were obtained from Santa Cruz Biotechnology. Antibodies against Flag tag (M2, A2220) and Wee1 (SAB4503088) were from Sigma. Antibodies against HA tag (C29F4) and p21 (2947S) were from Cell Signaling Technology. MLN4924 was purchased from Med Chem Express. Cycloheximide (CHX), dexamethasone (DEX), Trizol, methylthiazolyldiphenyl-tetrazolium bromide (MTT) and propidium iodide (PI) were purchased from Sigma.

### Tumor xenografts in mice

Nude mice at 4-6 weeks of age were purchased from SLAC Animal Center (Shanghai, China). MG63 cells (1 × 10^6^ in 200 μL of PBS) were injected subcutaneously near the scapula of the nude mice. 10 days after injection, the mice were randomly separated into two groups that received MLN4924 (30 mg/kg) or vehicle via intraperitoneal (i.p.) injection (twice a day for 3 days followed by no treatment for 2 days). Mice were euthanized after 15 days of the treatment and tumors were weighted. All animals were housed and handled in accordance with the institutional guide for the care and use of laboratory animals.

### RNA interference

The target sequences for the siRNAs against RORα, Bmal1, p21 and negative control (Control) siRNA were as following:

RORα-siRNA-1: 5′- GCCAAACGCAUUGAUGG AUTT -3′;

RORα-siRNA-2: 5′- GGAGUUGUUCACUUCAG AATT -3′;

Bmal1-siRNA-1: 5′- GGCCUUCAGUAAAGGUU GATT -3′;

Bmal1-siRNA-2: 5′- GGUGAAAUCUAUGGAAU AUTT-3′;

p21-siRNA-1: 5′- GAUGGAACUUCGACUUU GUTT-3′;

p21-siRNA-2: 5′-CCUCUGGCAUUAGAAUU AUTT-3′.

Control: 5′- UUCUCCGAACGUGCACGUTT -3′.

All siRNAs were purchased from Gene Pharma (Suzhou, China).

### Analysis of protein stability

U2OS cells were transfected with plasmids expressing Bmal1 or Flag-RORα using Lipofectamine 2000 reagent according to the manufacturer's protocol. At 48 h after transfection, cells were incubated with 0.1mg/mL cycloheximide (CHX) to inhibit protein synthesis. Cells were lysed with 2% SDS buffer (2% SDS, 150 mM NaCl, 10 mM Tris–HCl, pH 8.0, PMSF and protease inhibitor cocktail) at 0, 3, 6, 9 and 12 h after CHX treatment. Equal amounts of whole cell lysates were analyzed by Western blot with respective antibodies.

### Immunoprecipitation

U2OS cells were transfected with plasmids expressing Bmal1 or Flag-RORα and HA-tagged ubiquitin. After treatment, cells were lysed in 2% SDS buffer and boiled for 10 min followed by sonication. Lysates were diluted 1:10 in dilution buffer (10 mM Tris–HCl, pH 8.0, 150 mM NaCl, 2 mM EDTA, 1% Triton X-100), incubated at 4 °C for 1 h with rotation and centrifuged at 13000 g for 30 min. 500 μg of cell lysates were incubated with 1μg of antibody with constant agitation overnight at 4 °C. Then, 30 μL of protein-A agarose bead slurry was added to pull down the immune-complexes. Immunoprecipitated proteins were washed with washing buffer (10 mM Tris–HCl, pH 8.0, 1 M NaCl, 1 mM EDTA, 1% NP-40). After boiling with 2 × SDS loading buffer, the protein samples were analyzed by Western blot with respective antibodies.

### Real-time quantitative PCR (qPCR)

Total RNA was extracted using Trizol reagent according to the manufacturer's instruction. 2 μg of total RNA were reverse-transcribed using the RevertAid First Strand cDNA Synthesis Kit (Fermentas). The mRNA levels were examined with qPCR using 1 × SYBR Green PCR master mix (Fermentas) by C1000 touch Thermal Cycler (Bio-Rad). The primers were as following:

Actin-Forward: 5′-GGCATGGGTCAGAAGGATT-3′;

Actin-Reverse: 5′-TGGTGCCAGATTTTCTCCA-3′;

Bmal1-Forward: 5′-CCAAGAAAGTATGGACACAGACAAA-3′;

Bmal1-Reverse: 5′-GCATTCTTGATCCTTCCTTG GT-3′;

RORα-Forward: 5′-CAGGCTTCTTTCCCTACTGTTCGT-3′;

RORα-Reverse: 5′-CCGCTGCTTGTTTTGATAGTTCTC-3′.

### MTT assay

Cells were transfected with siRNAs targeting RORα, Bmal1 or p21, respectively. 24 h after transfection, cells were seeded in 96 well plates at the density of 1 × 10^3^ cells per well and incubated with MLN4924 (3 μM). After various times, the cells were stained with 20 μL of sterile MTT dye (5 mg/mL) for 4 h at 37 °C followed by removal of the culture medium and the addition of 200 μL of DMSO. The absorbance at 570 nm was measured and the absorbance at 630 nm was used as a reference. All assays were conducted at least three times and performed in triplicate on different days using different batches of cells.

### Flow cytometry

After treatment, cells were harvested and washed with cold PBS and fixed in ice-cold 70% ethanol overnight at −20°C, The fixed cells were incubated with 100 μL of propidium iodide (PI, 50 μg/mL) for 30 min at 4 °C in the dark. At least 10,000 cells were analyzed of the cell cycle using a Cytomics FC 500 instrument (Beckman Coulter) equipped with CXP software. To analyze apoptosis, cells were stained with Annexin V-FITC/PI, and apoptotic cells were examined by flow cytometry.

### Statistical analysis

All statistical analyses were performed using the Prism 6.01 (Graph Pad Software, San Diego, CA). Statistically significant differences between groups were determined by a repeated ANOVA followed by unpaired *t* test.

## SUPPLEMENTARY MATERIALS FIGURES


