# Supplementary material for: Circadian clock components RORα and Bmal1 mediate the anti-proliferative effect of MLN4924 in osteosarcoma cells
Source: Oncotarget. 2016 Sep 1;7(40):66087–99. doi: 10.18632/oncotarget.11807 (PMC5323217; doi:10.18632/oncotarget.11807)
Supplement: Supplementary file 1 [file oncotarget-07-66087-s001.pdf]

Circadian clock components RORα and Bmal1 mediate the anti-proliferative effect of MLN4924 in osteosarcoma cells

SUPPLEMENTARY FIGURES

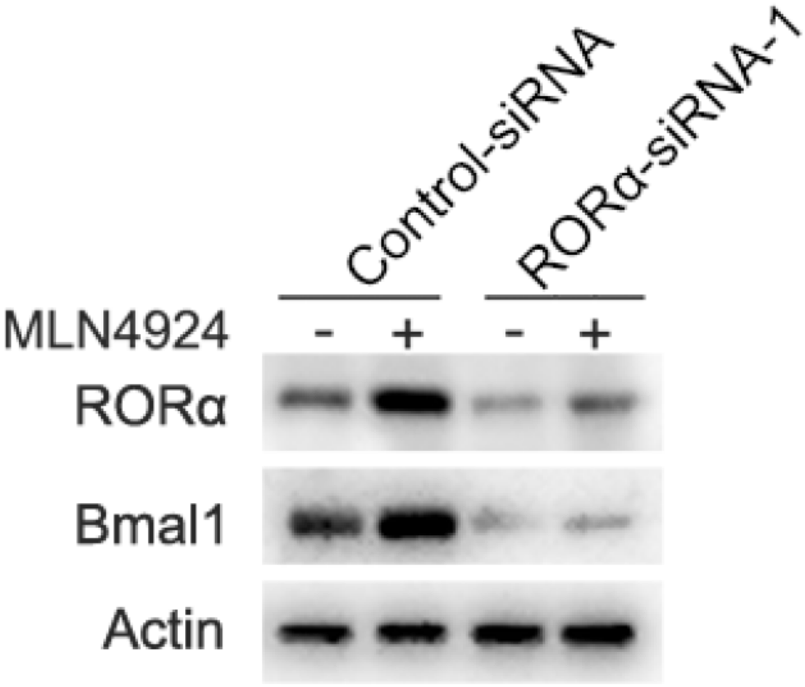

Supplementary Figure S1: Reduced Bmal1 levels in RORα-depleted U2OS cells. U2OS cells were treated with MLN4924 (1 μM) or DMSO 24 h after transfection with RORα-specific siRNA or control-siRNA, and the endogenous Bmal1 protein levels were detected with Western blot.

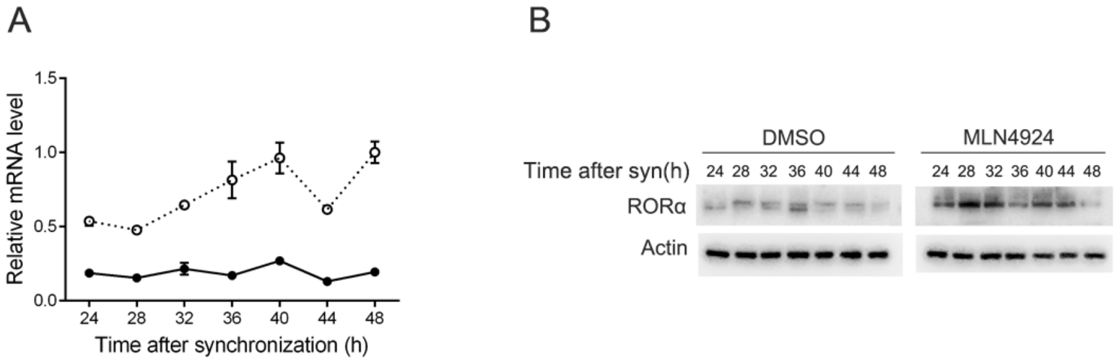

Supplementary Figure S2: MLN4924 increases RORα mRNA and protein levels in the synchronized U2OS cells. U2OS cells were synchronized with dexamethasone in the presence of MLN4924 (1 μM) or DMSO, and then harvested every 4 h beginning 24 h after synchronization. **A.** The RORα mRNA level was examined with qPCR using specific primers. The expression of RORα in cells treated with MLN4924 at 48 h was set as 1. Data from other treatments were presented as fold of this level. Each value is the mean ± SEM of three replicates from a single assay. **B.** The RORα protein level was examined with Western blot using specific antibody.

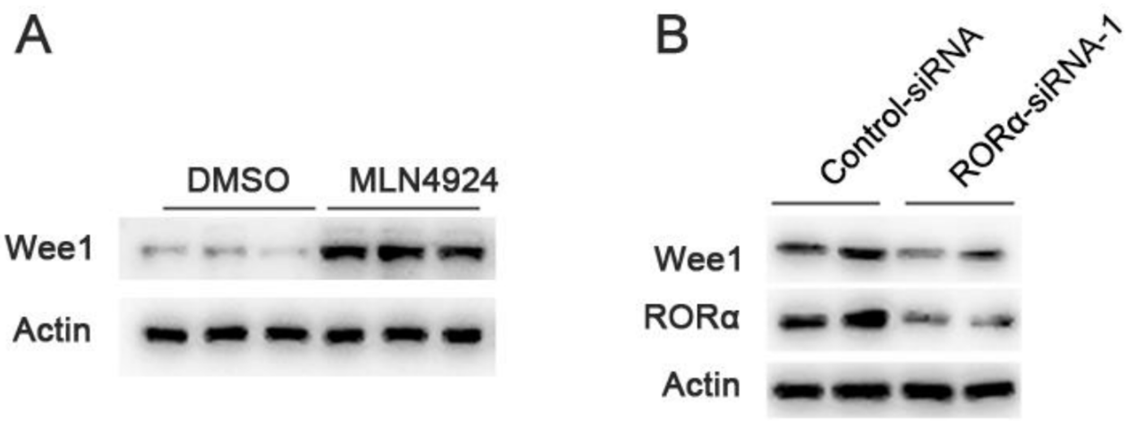

Supplementary Figure S3: MLN4924 increases Wee1 in U2OS osteosarcoma cells. A. The endogenous Wee1 protein levels were detected with Western blot after treatment with MLN4924 (1 μM) or DMSO in U2OS cells for 24 h. **B.** U2OS cells were transfected with RORα-specific siRNA1 or negative control siRNA. 72 h after transfection, whole cell lysates were analyzed with Western blot using antibodies for RORα, Wee1 and actin, respectively.
